# Supplementary material for: Real-time monitoring of multimode squeezing
Source: Nat Commun. 2026 Apr 29;17:3904. doi: 10.1038/s41467-026-72357-0 (PMC13129042; doi:10.1038/s41467-026-72357-0)
Supplement: Supplementary file 1 — Supplementary Information [file 41467_2026_72357_MOESM1_ESM.pdf]

Supplementary material to: Real-Time Monitoring of Multimode Squeezing

MAHMOUD KALASH, ADITYA SUDHARSANAM, M. H. M. PASSOS, VALENTINA PARIGI, AND MARIA CHEKHOVA

This supplementary document aims to support the reader in some specific points presented in the main manuscript.

1. DETAILED EXPERIMENTAL SCHEME

Figure S1 shows the full experimental setup. A 1 kHz, 18 ps, 70 μJ max energy per pulse, 354.67 nm laser is used to pump the nonlinear processes. The nonlinear medium employed is a 3 mm Bismuth Triborate (BiBO) crystal, cut for o->ee collinear degenerate parametric down-conversion (PDC). A lens L_0 focuses the pump inside the crystal with a Gaussian waist of 97 ± 2 μm, generating a spectrally and spatially multimode squeezed vacuum state (SV) in the direction of Pass 1. The gain of the squeezer is set to be $G_{sq} = 1.05 \pm 0.2$. The squeezer has a typical flat-top angular spectrum with FWHM about 20 mrad, as shown in Section 2, and 53 spatial modes resembling 2D Hermite-Gaussian distributions. A dichroic mirror (DM2) and a spherical mirror (SM) are used to reflect the SV back and image it one-to-one into the crystal in the direction of Pass 2. At the same time, the pump reflected from the PBS in the Pass 2 direction is imaged into the crystal using L2 and L3 with a waist of 145 μm. Its polarization is corrected using HWP2 to achieve o->ee phase matching for later phase-sensitive amplification using the same collinear-degenerate PDC. The pump power is adjusted to achieve an amplification gain of $G = 4.4 \pm 0.3$. Using the glass plate (P) we move the pump in the transverse plane in the crystal without altering

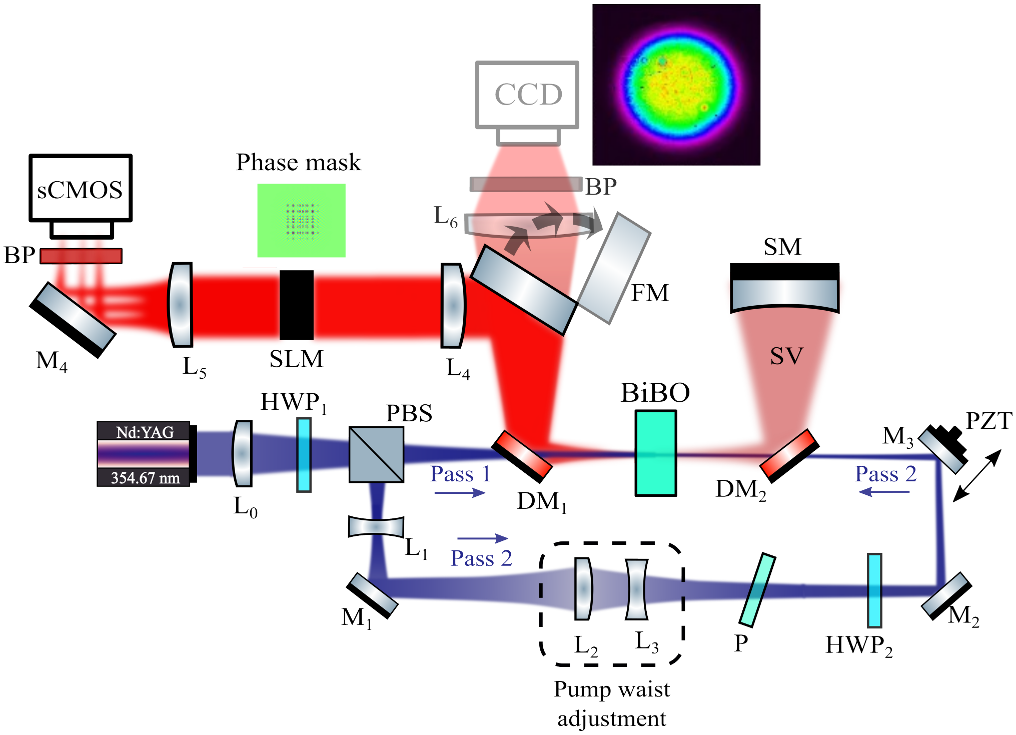

Fig. S1. Full experimental setup. L0-6, lenses; HWP1-2, half-wave plates; M1-4, mirrors; DM1-2, dichroic mirrors; PBS, polarizing beam splitter; P, glass plate; Pz, piezo actuator; SM, spherical mirror; SV, squeezed vacuum; BiBO, Bismuth Triborate crystal; BP, bandpass filter; FM, flip mirror; SLM, spatial light modulator; CCD, charge-coupled device camera.

the phase-matching, while tilting the SM modifies the direction of SV; both are used to achieve an alignment of the SV with the amplifier pump. Finally, the amplification phase is scanned using a piezo actuator (PZ) attached to mirror M3. DM1 is used to reflect the amplified radiation into the detection setup. With the flip mirror (FM) out, we image the far field of the radiation at the Fourier plane of a lens L_6 using a charge-coupled device (CCD) camera. A bandpass filter (BP) of 10 nm centered around the degenerate wavelength (709.33 nm) is used. When the mirror is flipped, the amplified radiation is then directed to the sorting setup. A system of three lenses with focal lengths $f_1 = 30$ cm, $f_2 = 40$ cm, and $f_3 = 30$ cm, respectively (denoted as compound lens L_4) images the amplified radiation onto the spatial light modulator (SLM, Hamamatsu LCOS-SLM X10468-06) positioned at the Fourier plane of lens L_4. Finally, an sCMOS camera, placed in the Fourier plane of lens L_5, monitors the intensities of the amplified modes.

2. THEORY OF THE MODAL CONTENT

For the theoretical calculation of the mode-dependent squeezing, mode-dependent amplification gain, and the modes of the source and the amplifier, we numerically solve the coupled integro-differential equations governing the collinear-degenerate parametric down-conversion process [1–3]. From the Bogoliubov transformations, we have

$$\hat{a}^{out}(q) = \int dq'\eta(q,q')\hat{a}^{in}(q') + \int dq'\beta(q,q')[\hat{a}^{in}(q')]^{\dagger}, \tag{S1}$$

with $\eta(q,q')$ and $\beta(q,q')$ are called the transfer functions and are related as

$$\begin{aligned} \frac{d\beta(q,q',L)}{dL} &= \Gamma \int dq'' F(q,q'')\eta^{*}(q'',q',L), \\ \frac{d\eta^{*}(q',q'',L)}{dL} &= \Gamma \int dq'' F^{*}(q,q'')\beta(q,q',L). \end{aligned} \tag{S2}$$

Here Γ is the coupling strength, and $F(q,q'') = \exp(-\frac{(q+q'')^2 w^2}{4})\exp(i\Delta k_z L)$ is related to the Gaussian pump transverse profile with waist w, the longitudinal phase-mismatch Δk_z, and crystal length L. Finally, the transfer functions, found through iterative calculations of Eqs(S2), can be decomposed into the Schmidt modes as

$$\begin{aligned} \beta(q,q') &= \sum_n \sqrt{\Lambda_n} u_n(q)\psi_n(q'), \\ \eta(q,q') &= \sum_n \sqrt{\Lambda'_n} u_n(q)\psi^{*}_n(q'), \end{aligned} \tag{S3}$$

with ψ_n/u_n being the modes at the input/output of the PDC process, $\Lambda_n = \sinh^2(G_n)$ define the mean photon number per mode n, and $\Lambda'_n = \Lambda_n + 1$.

2.1. Modes of the squeezer

For this calculation, we set L=3 mm, $w = 97\ \mu m$ and $G_{sq} = 1.05$. The latter, measured experimentally for the collinear emission, and corresponds in theory to $G_{sq} = b\Gamma$. The factor b can be found through fitting $N(q=0,\Gamma)$ with $a\sinh^2(b\Gamma)$, where $N(q=0,\Gamma) = \int dq'|\beta(q,q',\Gamma)|^2$ can be found by calculating β for different Γ. The results summarizing the modal content of the squeezer are shown in Fig.S2. In Fig.S2a the real part of $\beta(q,q')$ is shown, with the typical cross-correlation due to momentum conservation is observed. The 1D intensity distributions of the first (blue), second (brick), and third (yellow) modes are shown in Fig.S2b, they closely resemble the Hermite-Gauss modes with the waist of the fundamental mode being 23 μm. The normalized weights (i.e. $\sum_n \Lambda_n = 1$) corresponding to the strongest thirteen modes are presented in Fig.S2c. From the weights, the effective number of modes (also called the Schmidt number) can be calculated and amounts to $\nu = \frac{1}{\sum_n \Lambda_n^2} = 7.3$ modes in 1D, which corresponds to a total number of $\nu^2 = 53.3$ modes in 2D, assuming symmetric emission in x and y directions. Finally, the squeezing parameters $G_{sq,n}$ obtained from Λ_n values, are used to calculate the mode-dependent squeezing $Sq_n = 10\mathrm{Log}(\mathrm{e}^{-2G_{sq,n}})$ which are both shown in Fig.S2d. The squeezing parameters for 2D modes can be found from the relation between the normalized weights of these modes and the gain measured for collinear emission [4]. For 1D modes, we have $G_{sq,n} = \sqrt{\lambda_n} G_{sq}$. For 2D modes, we have $\lambda_{m,n} = \lambda_m \lambda_n$, then $G_{sq,mn} = \sqrt{\lambda_{m,n}} G_{sq}$. Finally, the squeezing parameters of 2D modes can be written in terms of the squeezing parameters of 1D modes as $G_{sq,mn} = \frac{G_{sq,m} G_{sq,n}}{G_{sq}}$.

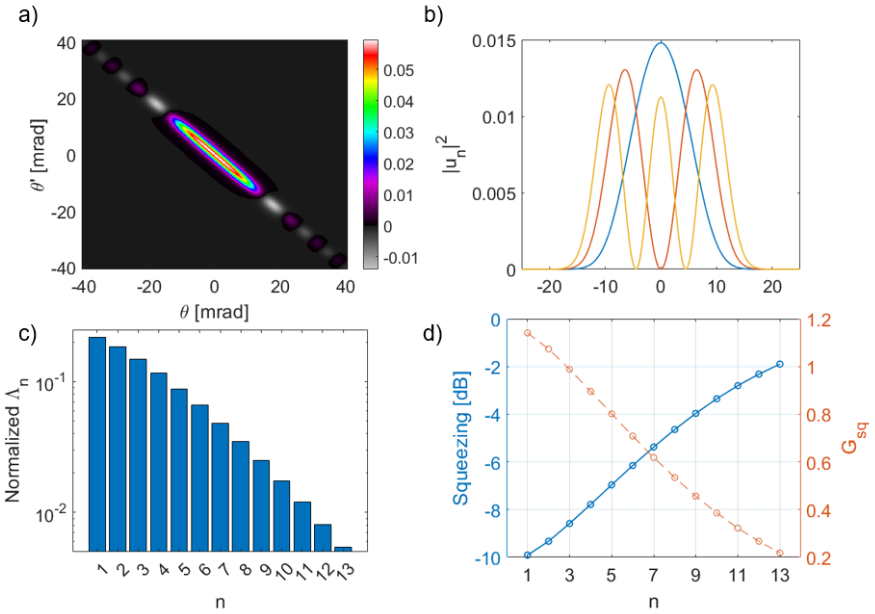

Fig. S2. Modal content of the squeezer. a) Real component of $\beta(q, q')$ calculated for L=3 mm, $w = 97\ \mu m$ and $G_{sq} = 1.05$. b) Intensity distributions of the first (blue), second (brick), and third (yellow) modes of the amplifier. c) Normalized weights of the thirteen strongest 1D modes. d) The squeezing parameters (brick) and the squeezing expected in the absence of losses (blue) of the thirteen strongest 1D modes.

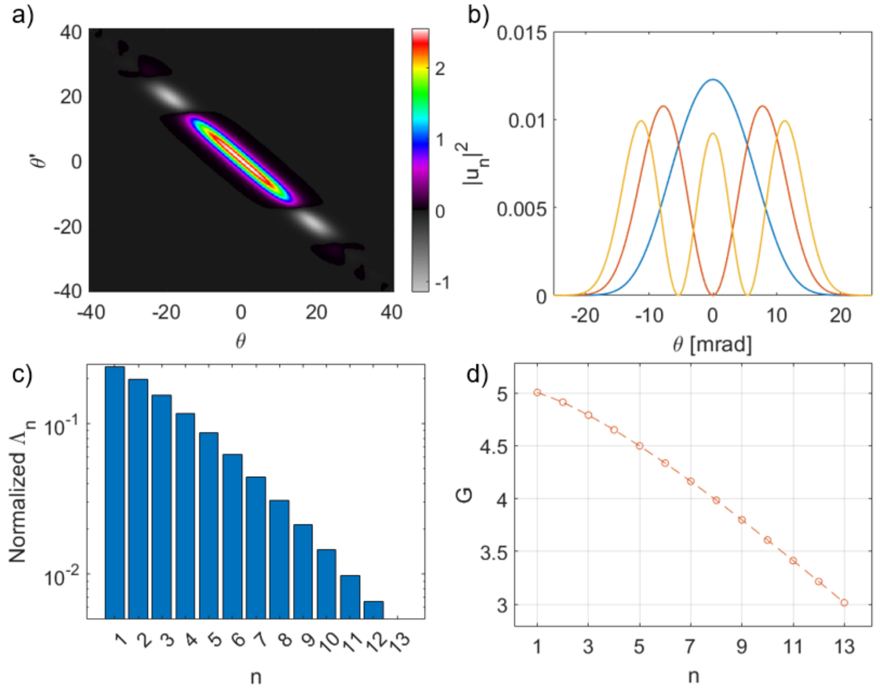

Fig. S3. Modal content of the MOPA. a) Real component of $\beta(q, q')$ calculated for L=3 mm, $w = 145\ \mu m$ and $G = 4.4$. b) Intensity distributions of the first (blue), second (brick), and third (yellow) modes of the amplifier. c) Normalized weights of the thirteen strongest 1D modes. d) Amplification gain G_n associated with the thirteen strongest 1D modes.

2.2. Modes of the MOPA

The same calculation is applied to the amplifier case, but the parameters $w = 145\ \mu m$ and $G = 4.4$ are used. Note that since now the pump waist is different, another round of calculations is needed to find the factor b. Fig. S3 shows the results for a) $\beta(q, q')$, b) 1D intensity distributions of the strongest three modes, and c) and d), the normalized weights and the amplification gains of the thirteen strongest modes respectively. The waist of the fundamental Gaussian mode now amounts to 19 μm, whereas the 1D Schmidt number reduces to 6.8 modes; i.e. 46.2 modes in 2D.

2.3. Mode Matching

The mode-matching figure presented in Fig. 2(a) of the main text shows nearly a diagonal distribution but with non-identity values. This is because the set of amplifier modes considered corresponds only to those measured in the experiment, and the non-identity values mean that the squeezer modes get overlapped with other higher-order amplifier ones. In order to find the complete overlap matrix, other amplifier modes are then considered. Figure S4 shows the overlap matrix between the output modes of the squeezer and 25 input modes of the amplifier, considering the experimental parameters. This matrix provides the full information because each squeezer mode is now fully decomposed into the considered amplifier modes.

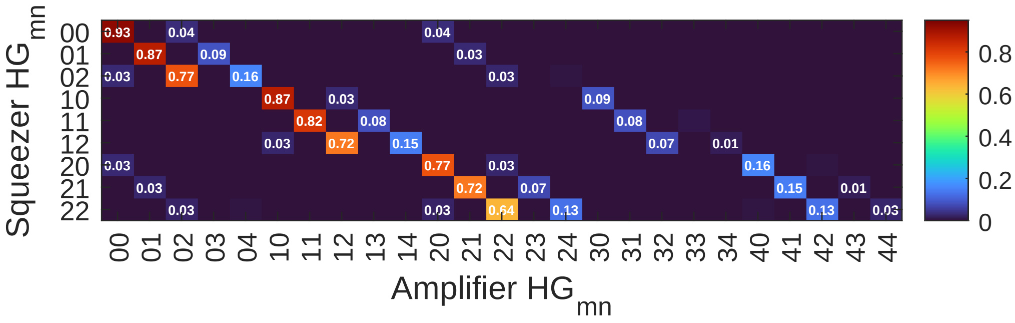

Fig. S4. Mode matching between the output modes of the squeezer and the input modes of MOPA calculated considering the pump waist of the squeezer as 97 μm while the pump of the amplifier has a waist of 145 μm, with $G_{sq} = 1.05$, $G = 4.4$.

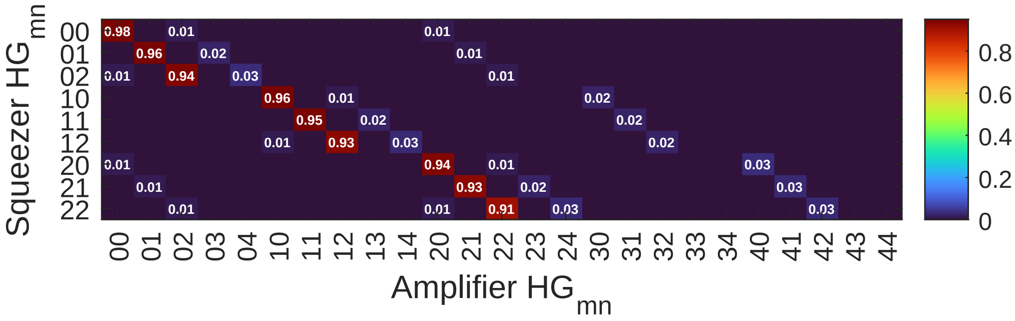

Fig. S5. Calculated mode matching for the case where the amplifier pump waist is 300 μm, while the other parameters are the same.

Figure S5 shows an example of calculated mode-matching coefficients when the amplifier pump is set to be 300 μm, while keeping the same other experimental parameters. The calculation shows that by doubling the size of the amplifier pump, mode matching is dramatically enhanced, especially for the higher-order modes. However, this can be experimentally demanding, since achieving the same amplification gain of $G = 4.4$ would require four times more pump power compared to the previous case. Since the choice of $G = 4.4$ is not strict, it can be set lower to suit the experimental context, making pump shaping a more flexible procedure. Mode matching can also be improved by differently imaging the squeezer modes into the amplifier; here, however, our goal is to demonstrate the proof-of-principle that pump shaping can strongly enhance the measurement of squeezing.

But even with imperfect mode matching, higher-order modes of the MOPA overlapping with the targeted mode of the squeezer do not affect the measurement result considerably. Figure S6 shows the squeezing and antisqueezing that would be measured for each mode in the ideal case (empty circles), in the presence of mode coupling without losses (black stars) and with 8% losses (blue stars). We see that losses affect the measurement much stronger than the coupling with other modes. The latter leads to only about 1 dB reduction of squeezing for mode HG_{22}, and even less for lower-order modes. The figure also shows experimental results (points with error bars). The deviation of these data from theoretical values for higher-order modes must be caused by imperfect alignment.

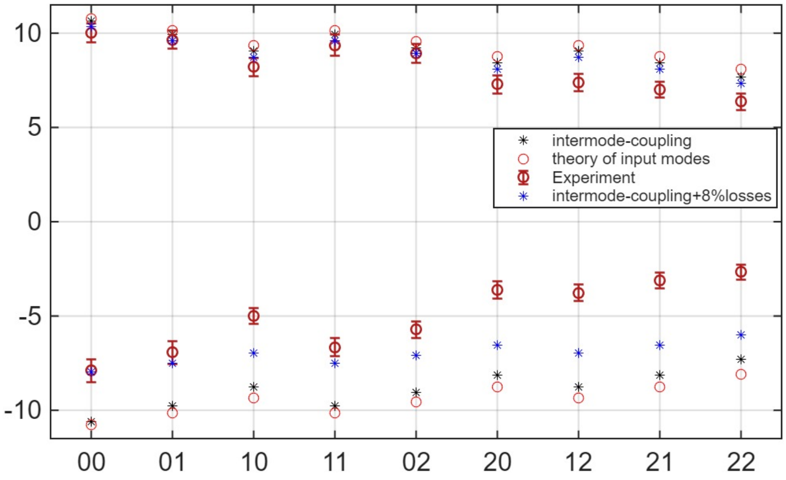

Fig. S6. The effect of mode coupling on the squeezing and antisqueezing measurement. Experimentally measured degrees of squeezing and antisqueezing for nine modes HG_{mn} (points with error bars), their theoretical values (empty circles), theoretical values accounting for the contribution of other modes (black stars), and with additional 8% losses (blue stars). Error bars correspond to standard deviation.

2.4. Accuracy of the measurement

Consider a squeezed vacuum state, with the squeezing parameter G_{sq}. After the amplification of quadrature $x^{(\phi)}$ with the amplification gain G, Eq. (1) of the main text requires the condition

$$e^{2G}\mathrm{Var}(x^{(\phi)}) \gg |e^{-2G}\mathrm{Var}(p^{(\phi)}) - \frac{1}{2}|, \tag{S4}$$

where $p^{(\phi)}$ is the conjugate quadrature of $x^{(\phi)}$. To be valid for any ϕ, this condition has to be satisfied for the case where the squeezed quadrature is amplified, i.e, $\phi = \pi/2$. Condition (S4) then becomes $e^{2(G-G_{sq})}\mathrm{Var}(x_{vac}) \gg |e^{-2(G-G_{sq})}\mathrm{Var}(p_{vac}) - \frac{1}{2}|$, with $\mathrm{Var}(x_{vac}) = \mathrm{Var}(p_{vac}) = \frac{1}{4}$. For $G - G_{sq} \gg 1$, the first term on the right is negligible, and the condition becomes $e^{2(G-G_{sq})} \gg 2$. The higher G, the lower the contribution of the conjugate quadrature to the measured photon number, and so the more accurate is the measurement. For instance, $e^{2(G-G_{sq})} \geq 20$ can be achieved with $G - G_{sq} \geq 1.5$. Still, at a finite amplification gain, the measurement is only accurate to a certain degree, due to the contribution of the conjugate quadrature to the measured mean intensity. The relative systematic inaccuracy in the measurement can be found as

$$\delta = \left| \frac{I_{app}^{(\phi)} - I^{(\phi)}}{I_{app}^{(\phi)}} \right|, \tag{S5}$$

where the approximate value $I_{app}^{(\phi)} = e^{2G}\langle [x^{(\phi)}]^2 \rangle$ has contribution only from the amplified quadrature and the accurate value for the intensity is $I^{(\phi)} = e^{2G}\langle [x^{(\phi)}]^2 \rangle + e^{-2G}\langle [p^{(\phi)}]^2 \rangle - \frac{1}{2}$.

For the accuracy of the squeezing measurement of an individual mode to be assessed, the modal gains of the squeezer and the amplifier should both be addressed individually. In Fig S7a, the modal gains are shown for the squeezer (blue) and the amplifier (brick). Given the calculated $G_{m,n}$ and $G_{sq,m,n}$, Fig S7.(b) shows that the inaccuracy of the squeezing measurement of an individual mode ranges between 0.027 % for the fundamental mode and 0.038 % for the HG22 mode, and it proves the quality of the measurement scheme.

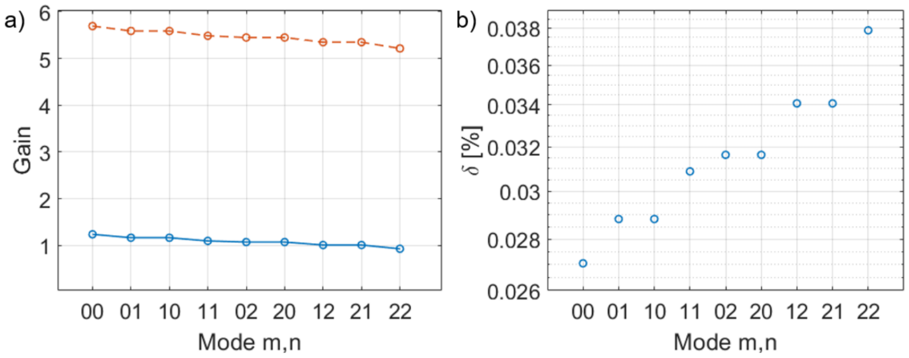

Fig. S7. a) $G_{sq,m,n}$ in blue and $G_{m,n}$ in brick. b) Relative inaccuracy of the squeezing measurement of different modes.

3. EXPERIMENTAL RECONSTRUCTION OF 2D MODES

3.1. Spatial intensity covariance measurement

The reconstruction of the amplifier's Schmidt modes involves measuring the PDC radiation's spatial intensity covariance. To calculate the covariance, we measure 1250 frames of the far-field

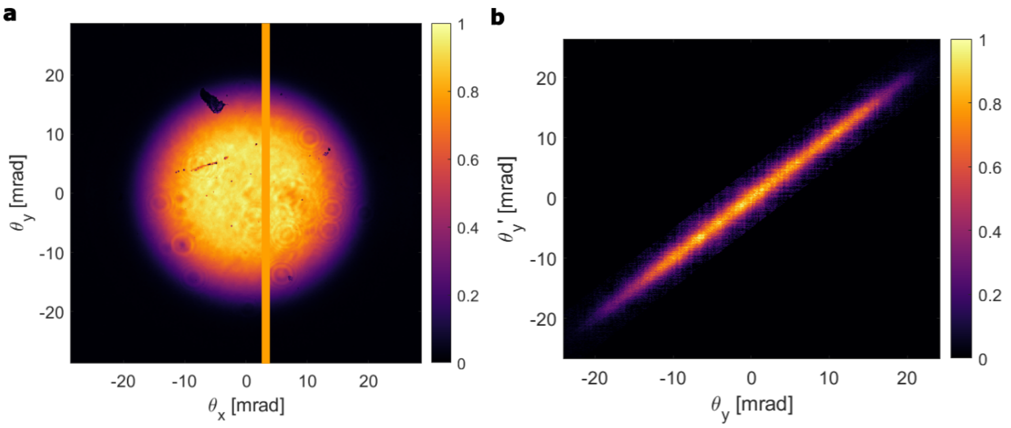

Fig. S8. (a) Far-field intensity of PDC from the amplifier averaged over 1250 frames. The orange and blue boxes indicate non-degenerate regions for which the covariance was measured. (b) Spatial intensity covariance along the y direction (orange).

intensity of PDC radiation (averaged far-field intensity distribution shown in Fig. S8(a)). The two-dimensional modes of PDC are factorable in the case of collinear-degenerate PDC [5], and hence the covariance can be calculated individually for x and y directions. The one-dimensional covariance calculation is ideally performed over a single column (row) of pixels.

$$\mathrm{Cov}(\theta_y, \theta_y') = \langle I(\theta_y) I(\theta_y') \rangle - \langle I(\theta_y) \rangle \langle I(\theta_y') \rangle, \tag{S6}$$

where $I(\theta_y)$ represents the intensity at point θ_y, and $\langle \cdots \rangle$ represents averaging over frames. However, a single column (row) is formed from averaging over 10 columns (rows) to reduce the effect of background intensity fluctuations. The orange stripe in Fig. S8(a) represents the

selected columns. The box is set off-center to account for the spatial symmetry in signal-idler pair generation, as this allows calculation of the purely autocorrelation of a single subsystem (signal or idler) of the PDC radiation. Fig. S8(b) presents the covariance matrix calculated across the columns. The same procedure performed across an off-center selection of rows yields the covariance along the x direction.

3.2. Two-dimensional Schmidt modes

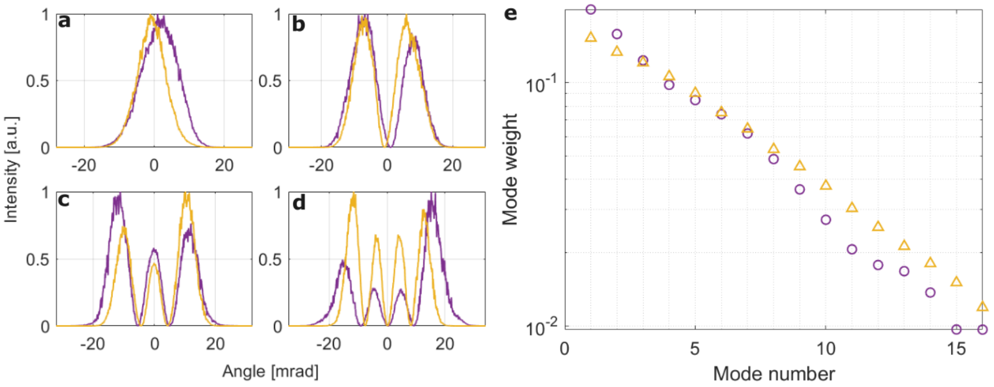

Fig. S9. (a) - (d) Comparison of 1D Schmidt modes reconstructed from covariance along the y (violet) and x directions (yellow). (e) Comparison of the reconstructed weights of the first 16 modes in the y (violet) and x (yellow) directions.

The auto-correlation of the one-dimensional intensity distribution can be decomposed via the singular value decomposition, which results in

$$\mathrm{Cov}(\theta_y, \theta_y') = \left(\sum_i \sqrt{\Lambda_i}\, u_m(\theta_y)\, u_m^*(\theta_y') \right)^2, \tag{S7}$$

where Λ_i represents the weights of the modes at high gain [4], and $u_m(\theta_y)$ is the one-dimensional Schmidt mode. The intensity distributions of the first four Schmidt modes obtained from the decomposition are presented in Fig. S9(a), and the normalised weights Λ_i of the first fifteen modes are shown in Fig. S9(b).

The reconstruction of two-dimensional modes $U_{m,n}(\theta_x, \theta_y) = u_m(\theta_x) \cdot u_n(\theta_y)$ is achieved by simply taking the product of the corresponding one-dimensional modes. Similarly, the weights of the two-dimensional modes are given by the product of the one-dimensional mode weights $\Lambda_{m,n} = \Lambda_m \cdot \Lambda_n$. Fig. S10 depicts the reconstructed intensity distributions of the nine highest-weighted two-dimensional modes and their weights.

4. SPATIAL MODE SORTING

4.1. Projective mode sorting

The Schmidt modes of PDC are known to be approximated by the Hermite-Gaussian (HG) basis of modes $HG_{m,n}(x, y)$. Therefore, the input PDC field $S(x, y)$ can be decomposed into the HG basis as

$$S(x, y) = \sum_{n=0}^{\infty} \sum_{m=0}^{\infty} a_{m,n} HG_{mn}(x, y), \tag{S8}$$

where $a_{m,n} = \rho_{m,n} e^{i\theta_{m,n}}$ is the complex expansion coefficient with amplitude $\rho_{m,n}$ and inter-modal phase $\theta_{m,n}$. Measuring the coefficients $a_{m,n}$ enables one to infer the physical parameters of the PDC field $S(x, y)$. Measurement of the modal coefficients is achieved by projecting the multimode field onto the modes of the HG basis

$$\left| \int_x dx \int_y dy [HG_{mn}(x,y)]^* S(x,y) \right|^2 = |\rho_{m,n}|^2 . \tag{S9}$$

The projection measurement yields an intensity ($\rho_{m,n}^2$) proportional to each mode's power content, or weight. The projective measurement is experimentally implemented using a phase mask displaying the complex conjugate of the mode $HG_{m,n}(x, y)$.

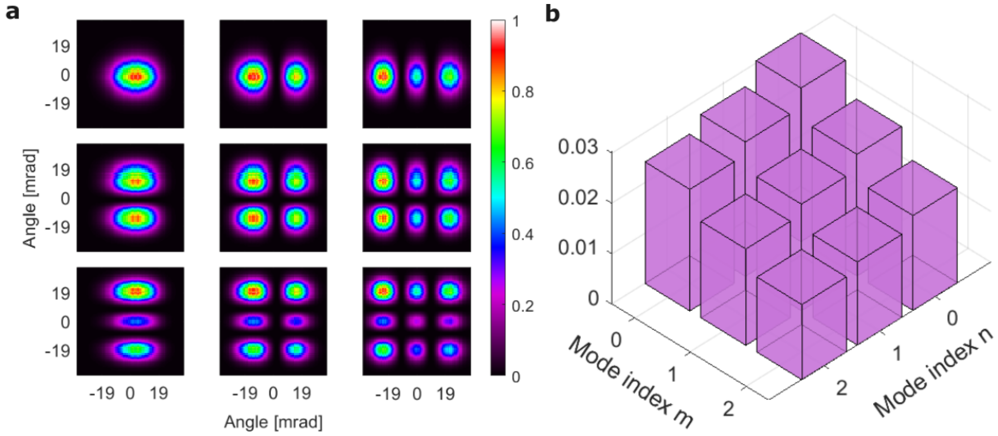

Fig. S10. (a) Reconstructed intensity of nine highest weighted 2D modes. (b) Corresponding mode weights.

4.2. Complex amplitude modulation

A phase-only SLM can be used to manipulate amplitude and phase by designing the phase mask using complex amplitude modulation. The procedure followed is adapted from the work of Arrizón et al. [6], and reproduced here for the reader's benefit.

The transverse spatial mode to be sorted is defined as

$$E(x, y) = E_0\, u(x, y) \exp[i\psi(x, y)], \tag{S10}$$

where E_0 is the magnitude of the electric field, $u(x, y) \in [0, 1]$ is the amplitude of the electric field, and $\psi(x, y) \in [-\pi, \pi]$ indicates its phase at the coordinates (x, y). The transmission function of the mask is described by

$$f(x, y) = \exp[i\theta(u, \psi)], \tag{S11}$$

where $\theta(u, \psi)$ denotes the phase of the mask, and is a function of the amplitude $u(x, y)$ and phase $\psi(x, y)$. One possible formulation of the phase mask $\theta(u, \psi)$ is given by [6]

$$\theta(u, \psi) = g(u) \sin(\psi), \tag{S12}$$

where the function $g(u)$ is an implicit function of (x, y) and encodes the amplitude information of the field $E(x, y)$. The authors of Ref. [6] show that an analytical solution to encoding amplitude information can be found using the Jacobi-Anger identity

$$\exp[ig(u) \sin(\psi)] = \sum_{p=-\infty}^{\infty} J_p[g(u)] \exp[ip\psi], \tag{S13}$$

where J_p is the Bessel function of the first kind of order p. It is therefore possible to encode the mask such that $E(x, y)$ is recovered in the term with $p = 1$. The amplitude information is encoded in the Bessel function $J_1[g(u)]$ and $g(u)$ is calculated by inverting the Bessel function $J_1[g(u)]$. $J_1(x)$ is an invertible function for $x \in [0, 1.84]$ where it attains a maximum of 0.582. The phase mask function $\theta(u, \psi)$ is therefore limited to a maximum phase range of 1.17π instead of 2π [6–8].

Mode sorting is completed when the contribution of the field $E(x, y)$ is spatially isolated from the incoming radiation. As the field is recovered in the term with $p = 1$, the terms of the expansion in Eq. (S13) must be spatially separated; this is achieved by modifying the phase $\psi(x, y)$ with a spatial diffraction grating $\psi'(x, y) = \psi(x, y) + 1.17\pi(\frac{x}{\Omega_x} + \frac{y}{\Omega_y})$, where Ω_x and Ω_y are constants denoting the spatial frequencies of the grating in the x and y directions, respectively.

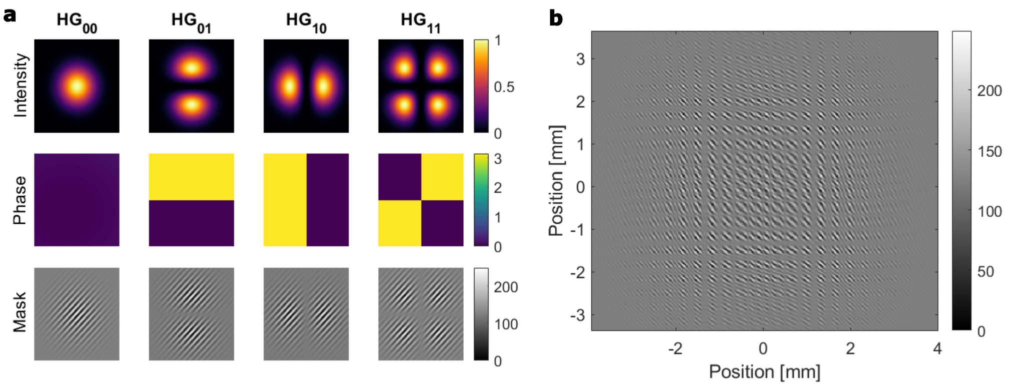

Fig. S11. (a) Top: Intensity distributions of lowest-order two-dimensional Hermite-Gaussian modes. Middle: Corresponding phase maps. Bottom: Phase mask encoded using Eq. (S15). (b) Phase mask encoded with modes $HG_{0,0}$ - $HG_{2,2}$ multiplexed using Eq. (S16).

4.3. Phase mask for Hermite-Gaussian modes

An HG mode of order m, n has the form [9]

$$\begin{aligned} HG_{m,n}(x,y,z) &= \sqrt{\frac{2^{(1-m-n)}}{\pi\, m!\, n!}}\, U_{m,n}(x,y,z)\, e^{[i\Phi_{m,n}(x,y,z)]}, \\ \text{with } U_{m,n}(x,y,z) &= \frac{1}{w(z)} \exp\left[-\frac{u(x,y)^2}{w(z)^2}\right] \times H_m\left(\frac{\sqrt{2}x}{w(z)}\right) H_n\left(\frac{\sqrt{2}y}{w(z)}\right), \\ \text{and } \Phi_{m,n}(x,y,z) &= \left[-kz + (1+n+m)\arctan\left(\frac{z}{z_R}\right)\right] - \left[\frac{ku(x,y)^2}{2R(z)}\right], \end{aligned} \tag{S14}$$

where λ is the wavelength and $k = \frac{2\pi}{\lambda}$ the wave vector. The parameters $w(z)$, $R(z)$, and z_R represent the waist, radius of curvature, and Rayleigh length of a Gaussian beam, respectively, and are defined as

$$w(z) = w_0\sqrt{1 + \left(\frac{z}{z_R}\right)^2},\ R(z) = z\left[1 + \left(\frac{z_R}{z}\right)^2\right], z_R = \frac{\pi w_0^2}{\lambda z^2},$$

with w_0 denoting the minimum beam waist. The intensity, phase, and phase masks of the first four HG modes are shown in Fig. S11(a).

To sort the mode $HG_{m,n}$, the amplitude $u(x, y)$ and phase $\psi(x, y)$ from Eq. (S12) are replaced by $U_{m,n}$ and $\Phi_{m,n}$. Additionally, the phase is modulated by a blazed diffraction grating, such that the sorted mode is directed into the first diffraction order at the output. The phase mask function, $\theta(x, y)$, is therefore written as

$$\begin{aligned} \theta(x,y) &= g_{HG}(x,y) \cdot \sin\left(\Phi_{m,n}(x,y) + 1.17\pi\left(\frac{x}{\Omega_x} + \frac{y}{\Omega_y}\right)\right), \\ \text{with } g_{HG}(x,y) &= J_1^{-1}\left(0.582 \cdot U_{m,n}(x,y)\right). \end{aligned} \tag{S15}$$

The masks generated using this formulation are displayed in the bottom row of Fig. S11(a). A mask multiplexing the modes $HG_{0,0}$ - $HG_{M,N}$ (Fig. S11b) is then encoded as [8]

$$\begin{aligned} \theta_{\text{mult}}(x,y) = \quad & \bmod\left\{\sum_{m=0}^{M}\sum_{n=0}^{N} J_1^{-1}\left(0.582 U_{mn}(x,y)\right) \sin\left(\Phi_{mn}(x,y)\right.\right. \\ & \left.\left. + 1.17\pi\left(\frac{x}{\Omega_x} + \frac{y}{\Omega_y}\right)\right), 1.17\pi\right\}. \end{aligned} \tag{S16}$$

4.4. Experimental implementation

Projective mode sorting is implemented using a phase-only SLM (Hamamatsu LCOS-SLM X10468-06) with a resolution of 20 μm per pixel. After phase-sensitive amplification, the far field of the

radiation is imaged onto the SLM using a system of lenses ($f_1 = 30$ cm, $f_2 = 40$ cm, $f_3 = 30$ cm), resulting in a beam with the FWHM of the fundamental mode 1.25 mm. The phase masks are encoded as described in [6] due to the high signal-to-noise ratio this method provides [7]. Simultaneous sorting of nine modes is achieved by multiplexing their holograms, so that the hologram of each mode is overlaid with a grating of a different spatial frequency [8]. An sCMOS camera (Andor Zyla 5.5) placed in the Fourier plane of a lens ($f_4 = 30$ cm) detects the projections of nine strongest Schmidt modes present in the incident radiation. The projections form a grid in the detection plane, allowing the intensity contribution (weight) of individual modes to be inferred by measuring the relative brightness in the center of each sorted mode.

4.5. Mode sorting efficiency

To calculate the transmission efficiency of mode sorting, we compare the intensity of the sorted mode (measured in its center to neglect the contributions of residual modes) to that of the expected intensity of this mode without sorting. The latter is obtained based on its mode weight $\Lambda_{m,n}$ (from covariance). For instance, consider mode HG_{00}: from Fig. S10(b), the weight of this mode is $\Lambda_{0,0} = 3.04 \pm 0.04\%$. When SLM is off, it works as a reflective mirror: we use this configuration to calculate the intensity of the HG_{00} mode as $I_{0,0} = \Lambda_{00} I_t$ where I_t is the intensity of the full angular spectrum after the SLM. In the experiment, it is found to be $I_t = (1.45 \pm 0.02) \times 10^7$ counts, which leads to $I_{0,0} = (4.41 \pm 0.23) \times 10^5$ counts. Meanwhile, the intensity of the HG_{00} mode after sorting is measured by selecting a 3×3 grid of pixels in the center and is found to be $I_{sorted} = (2.29 \pm 0.11) \times 10^3$ counts. Therefore, the efficiency of sorting amounts to $\eta_s = I_{sorted}/I_{0,0} \approx 0.52 \pm 0.04\%$.

5. CLUSTER STATES

5.1. Theoretical cluster framework

In this section, we aim to introduce the basic concepts of cluster states. Each cluster state can be topologically represented in a graph composed of several nodes, as shown in Figure S12. In the following subsections, we would like to discuss in detail cases a) and c) even though the main equations for the cases b) and d) will also be presented in this supplementary.

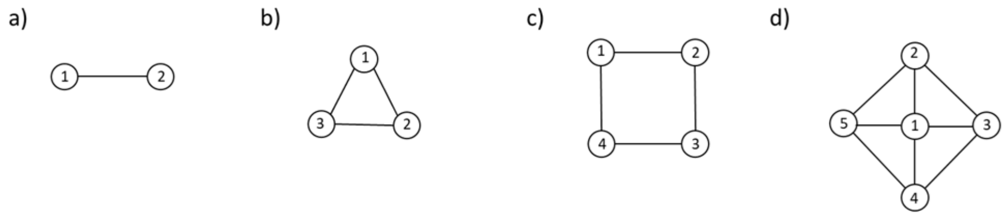

Fig. S12. Cluster topologies: a) Two nodes, b) Three nodes triangular, c) Four nodes squared shaped, and d) Five nodes.

5.2. Cluster quadratures

An N-mode squeezed vacuum consisting of N Schmidt modes can be described by the quadrature vector

$$\mathbf{q^s} = \{x_1^s, x_2^s, x_3^s, ..., x_N^s;\; p_1^s, p_2^s, p_3^s, ..., p_N^s\}, \quad \text{(S17)}$$

with x_i^s (p_i^s) being the position- (momentum-) like quadrature of the ith mode. The quadratures of the n-node cluster state to be obtained from the multimode squeezed vacuum,

$$\mathbf{Q^c} = \{X_1^c, X_2^c, X_3^c, ..., X_n^c;\; P_1^c, P_2^c, P_3^c, ..., P_n^c\}, \quad \text{(S18)}$$

are obtained by applying a unitary transformation U to the Schmidt-mode quadratures as

$$\mathbf{Q^c} = U\,\mathbf{q^s} = \begin{bmatrix} a & -b \\ b & a \end{bmatrix} \mathbf{q^s}, \quad \text{(S19)}$$

where

$$a = (\mathbb{1} + A^2)^{-1/2}, \quad \text{(S20)}$$

$$b = A\,(\mathbb{1} + A^2)^{-1/2}, \tag{S21}$$

and A is the adjacency matrix of each cluster topology.

Let us have closer look at two cases: a) Two-node linear cluster state (Fig. S12 - a)) and four-node square-shaped cluster state (Fig. S12 - c)).

Two-node linear cluster state: In this simplest case where we have a linear cluster composed of just two nodes, as can be seen in Figure S12.a), its adjacency matrix will be given by

$$A_{2,\text{linear}} = \begin{bmatrix} 0 & 1 \\ 1 & 0 \end{bmatrix}. \tag{S22}$$

In this way, by assuming the adjacency matrix described before together with the equations (S19), (S20), and (S21), one can directly obtain the cluster quadratures. For this case, they take the following forms:

$$X_1^c = \frac{x_1^s - p_2^s}{\sqrt{2}}, \tag{S23}$$

$$P_1^c = \frac{p_1^s + x_2^s}{\sqrt{2}} \tag{S24}$$

for node 1, and

$$X_2^c = \frac{x_2^s - p_1^s}{\sqrt{2}}, \tag{S25}$$

$$P_2^c = \frac{p_2^s + x_1^s}{\sqrt{2}} \tag{S26}$$

for node 2.

It is worth noticing that the cluster quadratures (X_i^c,P_i^c) for either node 1 or node 2 are always described in terms of the superposition of p-quadratures and x-quadratures of the first and the second Schmidt mode used to produce the two-node linear cluster state.

Four-node square-shaped cluster state: In this case the adjacency matrix takes the following form:

$$A_{4,\text{square}} = \begin{bmatrix} 0 & 1 & 0 & 1 \\ 1 & 0 & 1 & 0 \\ 0 & 1 & 0 & 1 \\ 1 & 0 & 1 & 0 \end{bmatrix}. \tag{S27}$$

Based on its adjacency matrix and following the same steps discussed in the previous case, the cluster quadratures for this four-node cluster state can be written as follows. For node 1, we have

$$X_1^c = \frac{1}{10}\left[-2\sqrt{5}\,p_2^s - 2\sqrt{5}\,p_4^s + \left(\sqrt{5}+5\right)\,x_1^s + \left(\sqrt{5}-5\right)\,x_3^s\right], \tag{S28}$$

$$P_1^c = \frac{1}{10}\left[\left(\sqrt{5}+5\right)\,p_1^s + \left(\sqrt{5}-5\right)\,p_3^s + 2\sqrt{5}\,(x_2^s + x_4^s)\right]. \tag{S29}$$

For node 2,

$$X_2^c = \frac{1}{10}\left[-2\sqrt{5}\,p_1^s - 2\sqrt{5}\,p_3^s + \left(\sqrt{5}+5\right)\,x_2^s + \left(\sqrt{5}-5\right)\,x_4^s\right], \tag{S30}$$

$$P_2^c = \frac{1}{10}\left[\left(\sqrt{5}+5\right)\,p_2^s + \left(\sqrt{5}-5\right)\,p_4^s + 2\sqrt{5}\,(x_1^s + x_3^s)\right]. \tag{S31}$$

For node 3,

$$X_3^c = \frac{1}{10}\left[-2\sqrt{5}\, p_2^s - 2\sqrt{5}\, p_4^s + \left(\sqrt{5}-5\right)\, x_1^s + \left(\sqrt{5}+5\right)\, x_3^s\right], \tag{S32}$$

$$P_3^c = \frac{1}{10}\left[\left(\sqrt{5}-5\right)\, p_1^s + \left(\sqrt{5}+5\right)\, p_3^s + 2\sqrt{5}\,(x_2^s + x_4^s)\right]. \tag{S33}$$

Finally, for node 4,

$$X_4^c = \frac{1}{10}\left[-2\sqrt{5}\, p_1^s - 2\sqrt{5}\, p_3^s + \left(\sqrt{5}-5\right)\, x_2^s + \left(\sqrt{5}+5\right)\, x_4^s\right], \tag{S34}$$

$$P_4^c = \frac{1}{10}\left[\left(\sqrt{5}-5\right)\, p_2^s + \left(\sqrt{5}+5\right)\, p_4^s + 2\sqrt{5}\,(x_1^s + x_3^s)\right]. \tag{S35}$$

As one might expect, the cluster quadratures (X_i^c,P_i^c) are now described in terms of superpositions of the x-quadratures and p-quadratures of the four Schmidt modes used to generate such cluster state.

5.3. Nullifiers Operators and Variances

Since we already have presented how to obtain a cluster state and hence their cluster quadratures, the natural next step is to check if we have a cluster state. It means that one must see if the nullifier operators are all squeezed. In other words, since we are not in the perfect scenario with infinite squeezing, the variance of the nullifier operators must be below the shot noise (SN) to guarantee that we have built a cluster state that could further be applied for quantum computational tasks, for instance. For clarity, let us again focus the discussions on the two previous examples from which we derived the cluster quadrature expressions. At the end of this subsection, we will also present the nullifier operators and variances for the other two cases (b) and d)) shown in Fig. S12. The normalized nullifier operators (δ_i) are defined as

$$\delta_i = \frac{\mathbf{P_i^c} - \sum_j^k A_{ij}\,\mathbf{X_j^c}}{\sqrt{1+n_i}}, \tag{S36}$$

where k is the number of nodes, n_i is the number of neighbors of node i, and A_{ij} are the matrix elements of the adjacent matrix A concerning the cluster state of interest.

Two-node linear cluster state: In this simplest case, as shown previously in Fig. S12 - a), our graph has just two nodes (labeled 1 and 2) so the way to obtain the pair of nullifier operators δ_1 and δ_2 is the following. Let us consider the first node, i.e, $i = 1$, which also has just one neighbor node ($n_1 = 1$). Following the equation (S36), we will find that

$$\delta_1 = \frac{P_1^c - \sum_{j=1}^2 A_{1j}\,X_j^c}{\sqrt{2}} = \frac{P_1^c - (\,A_{11}\,X_1^c + A_{12}\,X_2^c\,)}{\sqrt{2}}, \tag{S37}$$

$$\delta_1 = \frac{P_1^c - X_2^c}{\sqrt{2}}, \tag{S38}$$

since A_{11} is the first matrix element of the Eq. (S22) and it is equal to zero. Following the previous equation, one can see that the nullifier operator is described by a combination of the p and q quadratures of the cluster nodes. However, it can also be written in terms of the Schmidt modes by using the equations (S24) and (S25). Thus,

$$\delta_1 = \frac{P_1^c - X_2^c}{\sqrt{2}} = \frac{1}{\sqrt{2}}\left(\frac{p_1^s + x_2^s}{\sqrt{2}} - \frac{x_2^s - p_1^s}{\sqrt{2}}\right) = p_1^s. \tag{S39}$$

(S40)

In the same way, for the second node ($i = 2$), one has

$$\delta_2 = \frac{P_2^c - \sum_{j=1}^{2} A_{2j} X_j^c}{\sqrt{2}} = \frac{P_2^c - (A_{21} X_1^c + A_{22} X_2^c)}{\sqrt{2}} \tag{S41}$$

$$\delta_2 = \frac{P_2^c - X_1^c}{\sqrt{2}}, \tag{S42}$$

since $A_{22} = 0$, as can be seen in the Eq. (S22), and $n_2 = 1$. Following the same procedure as before and assuming the equations (S23) and (S26), one will obtain

$$\delta_2 = \frac{P_2^c - X_1^c}{\sqrt{2}} = p_2^s. \tag{S43}$$

Since we already have the expression for each nullifier in terms of the Schmidt modes, the next step is to calculate the variance of those operators based on equations (S39) and (S43). In this way, their variances $\Delta^2\hat{\delta}_1$ and $\Delta^2\hat{\delta}_2$ will be given by

$$\Delta^2\delta_1 = \left\langle \delta_1 \cdot \delta_1 \right\rangle = \left\langle \left(p_1^s \right)^2 \right\rangle \tag{S44}$$

$$\Delta^2\delta_2 = \left\langle \delta_2 \cdot \delta_2 \right\rangle = \left\langle \left(p_2^s \right)^2 \right\rangle. \tag{S45}$$

It is important to highlight that since the variances of the two nullifiers δ_1 and δ_2 are defined in terms of the variance of the p-quadrature of each Schmidt mode, it can be measure simultaneously by an OPA.

Four-node square-shaped cluster state: Another graph that is relevant to our discussions is the four nodes squared-shaped one (Fig S12.c)). In this case, we should obtain four nullifier operators, δ_1, δ_2, δ_3, and δ_4. The procedure is the same as before, except that in this graph, every node has two neighbors. Thus, for the first node ($i = 1$), hence the first nullifier operator, we have

$$\delta_1 = \frac{P_1^c - \sum_{j=1}^{4} A_{1j} X_j^c}{\sqrt{3}} = \frac{P_1^c - (A_{11} X_1^c + A_{12} X_2^c + A_{13} X_3^c + A_{14} X_4^c)}{\sqrt{3}}, \tag{S46}$$

$$= \frac{P_1^c - X_2^c - X_4^c}{\sqrt{3}} = \frac{\left(1+\sqrt{5}\right) p_1^s + \left(-1+\sqrt{5}\right) p_3^s}{2\sqrt{3}}. \tag{S47}$$

Here, A_{11} = A_{13}= 0, as can be seen in the Eq. (S27).

The second nullifier operator takes the form

$$\delta_2 = \frac{P_2^c - \sum_{j=1}^{2} 4\, A_{1j} X_j^c}{\sqrt{3}} = \frac{P_2^c - (A_{11} X_1^c + A_{12} X_2^c + A_{13} X_3^c + A_{14} X_4^c)}{\sqrt{3}}, \tag{S48}$$

$$= \frac{P_2^c - X_1^c - X_3^c}{\sqrt{3}} = \frac{\left(1+\sqrt{5}\right) p_2^s + \left(-1+\sqrt{5}\right) p_4^s}{2\sqrt{3}}, \tag{S49}$$

since A_{22} = A_{24}= 0 in the Eq. (S27).

The other two nullifiers δ_3 and $\hat{\delta}_4$ are written following the same procedure as described above. They have the forms

$$\delta_3 = \frac{P_3^c - X_2^c - X_4^c}{\sqrt{3}} = \frac{\left(-1+\sqrt{5}\right) p_1^s + \left(1+\sqrt{5}\right) p_3^s}{2\sqrt{3}}, \tag{S50}$$

$$\delta_4 = \frac{P_4^c - X_1^c - X_3^c}{\sqrt{3}} = \frac{\left(-1+\sqrt{5}\right) p_2^s + \left(1+\sqrt{5}\right) p_4^s}{2\sqrt{3}}. \tag{S51}$$

The variances of each nullifier are

$$\Delta^2\delta_1 = \left\langle \delta_1 \cdot \delta_1 \right\rangle = \left(\frac{\sqrt{5}+3}{6}\right) \left\langle \left(p_1^s\right)^2 \right\rangle - \left(\frac{\sqrt{5}-3}{6}\right) \left\langle \left(p_3^s\right)^2 \right\rangle \quad \text{(S52)}$$

$$= -7.74 \pm 0.54 \text{ dB}, \quad \text{(S53)}$$

$$\Delta^2\delta_2 = \left\langle \delta_2 \cdot \delta_2 \right\rangle = \left(\frac{\sqrt{5}+3}{6}\right) \left\langle \left(p_2^s\right)^2 \right\rangle - \left(\frac{\sqrt{5}-3}{6}\right) \left\langle \left(p_4^s\right)^2 \right\rangle \quad \text{(S54)}$$

$$= -6.78 \pm 0.53 \text{ dB}, \quad \text{(S55)}$$

(S56)

$$\Delta^2\delta_3 = \left\langle \delta_3 \cdot \delta_3 \right\rangle = -\left(\frac{\sqrt{5}-3}{6}\right) \left\langle \left(p_1^s\right)^2 \right\rangle + \left(\frac{\sqrt{5}+3}{6}\right) \left\langle \left(p_3^s\right)^2 \right\rangle \quad \text{(S57)}$$

$$= -6.82 \pm 0.41 \text{ dB}, \quad \text{(S58)}$$

$$\Delta^2\delta_4 = \left\langle \delta_4 \cdot \delta_4 \right\rangle = -\left(\frac{\sqrt{5}-3}{6}\right) \left\langle \left(p_2^s\right)^2 \right\rangle + \left(\frac{\sqrt{5}+3}{6}\right) \left\langle \left(p_4^s\right)^2 \right\rangle \quad \text{(S59)}$$

$$= -5.87 \pm 0.38 \text{ dB}. \quad \text{(S60)}$$

Three nodes (triangle) : The topology for this cluster state is shown in the figure S12 b). The nullifiers for the three nodes cluster state in a triangular shape will take the following form

$$\delta_1 = \frac{P_1^c - X_2^c - X_3^c}{\sqrt{3}} = 0.974663\ p_1^s + 0.158166\ p_2^s + 0.158166\ p_3^s, \quad \text{(S61)}$$

$$\delta_2 = \frac{P_2^c - X_1^c - X_3^c}{\sqrt{3}} = 0.158166\ p_1^s + 0.974663\ p_2^s + 0.158166\ p_3^s, \quad \text{(S62)}$$

$$\delta_3 = \frac{P_3^c - X_1^c - X_2^c}{\sqrt{3}} = 0.158166\ p_1^s + 0.158166\ p_2^s + 0.974663\ p_3^s, \quad \text{(S63)}$$

where we consider for this situation that each node has three neighbors $n_{1,2,3} = 2$.
The variances for this topology are

$$\Delta^2\delta_1 = 0.949967 \left\langle \left(p_1^s\right)^2 \right\rangle + 0.0250165 \left\langle \left(p_2^s\right)^2 \right\rangle + 0.0250165 \left\langle \left(p_3^s\right)^2 \right\rangle \quad \text{(S64)}$$

$$= -7.85 \pm 0.58 \text{ dB}, \quad \text{(S65)}$$

$$\Delta^2\delta_2 = 0.0250165 \left\langle \left(p_1^s\right)^2 \right\rangle + 0.949967 \left\langle \left(p_2^s\right)^2 \right\rangle + 0.0250165 \left\langle \left(p_3^s\right)^2 \right\rangle \quad \text{(S66)}$$

$$= -6.95 \pm 0.57 \text{ dB}, \quad \text{(S67)}$$

$$\Delta^2\delta_3 = 0.0250165 \left\langle \left(p_1^s\right)^2 \right\rangle + 0.0250165 \left\langle \left(p_2^s\right)^2 \right\rangle + 0.949967 \left\langle \left(p_3^s\right)^2 \right\rangle \quad \text{(S68)}$$

$$= -6.70 \pm 0.45 \text{ dB}. \quad \text{(S69)}$$

Five nodes: The topology for this cluster state is shown in the figure S12 d). The nullifiers for the five nodes cluster state will be slightly different from the previous case since the number of neighbors will not be the same for all of the nodes anymore. For instance, the first nullifier ($i = 1$) has $n_1 = 4$ meanwhile all the other nullifiers for this cluster will have three neighbors. Then,

$$\begin{aligned} \delta_1 &= \frac{P_1^c - X_2^c - X_3^c - X_4^c - X_5^c}{\sqrt{5}} \\ &= 0.933174\ p_1^s + 0.179713\ p_2^s + 0.179713\ p_3^s \\ &+ 0.179713\ p_4^s + 0.179713\ p_5^s \end{aligned} \quad \text{(S70)}$$

$$\begin{aligned}
\delta_2 &= \frac{P_2^c - X_1^c - X_3^c - X_5^c}{\sqrt{4}}, \\
&= 0.200925\ p_1^s + 0.890801\ p_2^s + 0.081784\ p_3^s \\
&+ 0.390801\ \hat{p}_4^s + 0.081784\ p_5^s
\end{aligned} \tag{S71}$$

$$\begin{aligned}
\delta_3 &= \frac{P_3^c - X_1^c - X_2^c - X_4^c}{\sqrt{4}} \\
&= 0.200925\ p_1^s + 0.081784\ p_2^s + 0.890801\ p_3^s \\
&+ 0.081784\ p_4^s + 0.390801\ p_5^s
\end{aligned} \tag{S72}$$

$$\begin{aligned}
\delta_4 &= \frac{P_4^c - X_1^c - X_3^c - X_5^c}{\sqrt{4}} \\
&= 0.200925\ p_1^s + 0.390801\ p_2^s + 0.081784\ p_3^s \\
&+ 0.890801\ p_4^s + 0.081784\ p_5^s
\end{aligned} \tag{S73}$$

$$\begin{aligned}
\delta_5 &= \frac{P_5^c - X_1^c - X_2^c - X_4^c}{\sqrt{4}} \\
&= 0.200925\ p_1^s + 0.081784\ p_2^s + 0.390801\ p_3^s \\
&+ 0.081784\ p_4^s + 0.890801\ p_5^s
\end{aligned} \tag{S74}$$

Hence, the variance of each nullifier will be given by

$$\begin{aligned}
\Delta^2\delta_1 &= 0.870813 \left\langle \left(p_1^s\right)^2 \right\rangle + 0.0322967 \left\langle \left(p_2^s\right)^2 \right\rangle + 0.0322967 \left\langle \left(p_3^s\right)^2 \right\rangle \\
&+ 0.0322967 \left\langle \left(p_4^s\right)^2 \right\rangle + 0.0322967 \left\langle \left(p_5^s\right)^2 \right\rangle && \text{(S75)} \\
&= -7.65\ \pm\ 0.53\ \text{dB}, && \text{(S76)} \\
\Delta^2\delta_2 &= 0.0403709 \left\langle \left(p_1^s\right)^2 \right\rangle + 0.793526 \left\langle \left(p_2^s\right)^2 \right\rangle + 0.00668863 \left\langle \left(p_3^s\right)^2 \right\rangle \\
&+ 0.152725 \left\langle \left(p_4^s\right)^2 \right\rangle + 0.00668863 \left\langle \left(p_5^s\right)^2 \right\rangle && \text{(S77)} \\
&= -6.77\ \pm\ 0.48\ \text{dB}, && \text{(S78)} \\
\Delta^2\delta_3 &= 0.0403709 \left\langle \left(p_1^s\right)^2 \right\rangle + 0.00668863 \left\langle \left(p_2^s\right)^2 \right\rangle + 0.793526 \left\langle \left(p_3^s\right)^2 \right\rangle \\
&+ 0.00668863 \left\langle \left(p_4^s\right)^2 \right\rangle + 0.152725 \left\langle \left(p_5^s\right)^2 \right\rangle && \text{(S79)} \\
&= -6.42\ \pm\ 0.38\ \text{dB}, && \text{(S80)} \\
\Delta^2\delta_4 &= 0.0403709 \left\langle \left(p_1^s\right)^2 \right\rangle + 0.152725 \left\langle \left(p_2^s\right)^2 \right\rangle + 0.00668863 \left\langle \left(p_3^s\right)^2 \right\rangle \\
&+ 0.793526 \left\langle \left(p_4^s\right)^2 \right\rangle + 0.00668863 \left\langle \left(p_5^s\right)^2 \right\rangle && \text{(S81)} \\
&= -6.00\ \pm\ 0.35\ \text{dB}, && \text{(S82)} \\
\Delta^2\delta_5 &= 0.0403709 \left\langle \left(p_1^s\right)^2 \right\rangle + 0.00668863 \left\langle \left(p_2^s\right)^2 \right\rangle + 0.152725 \left\langle \left(p_3^s\right)^2 \right\rangle \\
&+ 0.00668863 \left\langle \left(p_4^s\right)^2 \right\rangle + 0.793526 \left\langle \left(p_5^s\right)^2 \right\rangle && \text{(S83)} \\
&= -5.34\ \pm\ 0.32\ \text{dB}. && \text{(S84)}
\end{aligned}$$

5.4. Cluster modes and nullifier modes

For the sake of clarity, we would like to provide the reader a step further on the physical intuition about the cluster states. In this way, we would like to merge this subsection with the previous concept that we introduced in the previous subsections and comment on the cluster modes and the nullifier modes. Here, let us consider again the simplest case of two-node cluster states.

The cluster modes ($M_i^{cluster}$) are obtained based on the unitary transformation

$$\begin{aligned} u_{modes} &= a + i\, b \\ &= \left((\mathbb{1} + A^2)^{-1/2} + i\, A\, (\mathbb{1} + A^2)^{-1/2} \right) \\ &= \begin{bmatrix} \frac{1}{\sqrt{2}} & \frac{i}{\sqrt{2}} \\ \frac{i}{\sqrt{2}} & \frac{1}{\sqrt{2}} \end{bmatrix}. \end{aligned} \tag{S85}$$

Thus, one can write a mode vector m_{OPA} composed of the N Schmidt modes. For this simple example, N is equal to two, i.e., two Schmidt modes equally squeezed, such as, for instance, HG_{01} and HG_{10}. In this way,

$$M_i^{cluster} = \begin{bmatrix} \frac{1}{\sqrt{2}} & \frac{i}{\sqrt{2}} \\ \frac{i}{\sqrt{2}} & \frac{1}{\sqrt{2}} \end{bmatrix} m_i^{OPA} \tag{S86}$$

$$\begin{bmatrix} M_1^{cluster} \\ M_2^{cluster} \end{bmatrix} = \begin{bmatrix} \frac{1}{\sqrt{2}} & \frac{i}{\sqrt{2}} \\ \frac{i}{\sqrt{2}} & \frac{1}{\sqrt{2}} \end{bmatrix} \begin{bmatrix} HG_{01} \\ HG_{10} \end{bmatrix} \tag{S87}$$

$$\begin{bmatrix} M_1^{cluster} \\ M_2^{cluster} \end{bmatrix} = \begin{bmatrix} \frac{HG_{01} + i\, HG_{10}}{\sqrt{2}} \\ \frac{i\, HG_{01} + HG_{10}}{\sqrt{2}} \end{bmatrix}. \tag{S88}$$

Based on the previous equations, it is straightforward to see that the first ($i = 1$) and second ($i = 2$) cluster mode are coherent superpositions of the two Hermite Gaussian modes of the amplifier. In other words, the cluster modes are Laguerre-Gaussian modes, for instance, $M_1^{cluster} = LG_{1,0}$ and $M_2^{cluster} = i\, LG_{-1,0}$, with the clockwise and anti-clockwise same azimuthal number ($l = 1$). This example is illustrated in Fig. 4 of our manuscript.

Normalized nullifier modes (M_i^{Null}), where i is the ith nullifier mode, can be obtained by the following unitary should

$$M_i^{Null} = [Diag(r1, r2, ...)(\mathbb{1} - iA)]^{\dagger} M_i^{cluster}, \tag{S89}$$

where $r_i = \sqrt{1 + n_i}$ is the normalizing factor related to the number of the nearest neighbor n_i of node i.

For the simplest case of two-node clusters, where $n_{1,2} = 1$, we obtain

$$M_i^{null} = \begin{bmatrix} \sqrt{2} & i\sqrt{2} \\ i\sqrt{2} & \sqrt{2} \end{bmatrix} M_i^{cluster} \tag{S90}$$

$$\begin{bmatrix} M_1^{null} \\ M_2^{null} \end{bmatrix} = \begin{bmatrix} \sqrt{2} & i\sqrt{2} \\ i\sqrt{2} & \sqrt{2} \end{bmatrix} \begin{bmatrix} M_1^{cluster} \\ M_2^{cluster} \end{bmatrix} \tag{S91}$$

$$\begin{bmatrix} M_1^{null} \\ M_2^{null} \end{bmatrix} = \begin{bmatrix} \frac{M_1^{cluster} + i\, M_2^{cluster}}{\sqrt{2}} \\ \frac{i\, M_1^{cluster} + M_2^{cluster}}{\sqrt{2}} \end{bmatrix}. \tag{S92}$$

By replacing the cluster modes with expressions obtained in the equation S88, one can write the nullifier modes in terms of the Schmidt modes. Thus

$$M_1^{null} = 2iHG_{10}, \tag{S93}$$

$$M_2^{null} = 2iHG_{01}. \tag{S94}$$

These expressions illustrate the nullifier modes presented in Figure 4.d - (ii) of the main manuscript.

REFERENCES

1. A. Christ, B. Brecht *et al.*, "Theory of quantum frequency conversion and type-II parametric down-conversion in the high-gain regime," New J. Phys. **15**, 053038 (2013).
2. P. R. Sharapova, G. Frascella *et al.*, "Properties of bright squeezed vacuum at increasing brightness," Phys. Rev. Res. **2**, 013371 (2020).
3. D. Scharwald, T. Meier *et al.*, "Phase sensitivity of spatially broadband high-gain $SU(1,1)$ interferometers," Phys. Rev. Res. **5**, 043158 (2023).
4. P. Sharapova, A. M. Pérez *et al.*, "Schmidt modes in the angular spectrum of bright squeezed vacuum," Phys. Rev. A **91**, 043816 (2015).
5. G. Frascella, R. V. Zakharov *et al.*, "Experimental reconstruction of spatial Schmidt modes for a wide-field SU(1,1) interferometer," Laser Phys. **29**, 124013 (2019).
6. V. Arrizón, U. Ruiz, R. Carrada, and L. A. González, "Pixelated phase computer holograms for the accurate encoding of scalar complex fields," J. Opt. Soc. Am. A **24**, 3500–3507 (2007).
7. T. W. Clark, R. F. Offer, S. Franke-Arnold, A. S. Arnold, and N. Radwell, "Comparison of beam generation techniques using a phase only spatial light modulator," Opt. Exp. **24**, 6249–6264 (2016).
8. C. Rosales-Guzmán, N. Bhebhe, and A. Forbes, "Simultaneous generation of multiple vector beams on a single SLM," Opt. Exp. **25** (2017).
9. B. E. A. Saleh and M. C. Teich, *Fundamentals Of Photonics* (John Wiley & Sons, Inc., 2019).
